# Persistent Helicobacter pylori Infection: An Insight to the Limitations of Current Clinical Practice

**DOI:** 10.7759/cureus.12309

**Published:** 2020-12-26

**Authors:** Anabel Liyen Cartelle, Pearl Princess Uy, Tara E Koehler, John Erikson L Yap

**Affiliations:** 1 Department of Medicine, Medical College of Georgia - Augusta University, Augusta, USA; 2 Gastroenterology and Hepatology, Medical College of Georgia - Augusta University, Augusta, USA; 3 Internal Medicine: Gastroenterology, Augusta University Medical Center, Augusta, USA

**Keywords:** helicobacter pylori, refractory infection, rifabutin triple therapy, patient education

## Abstract

The discovery of the pathological role of *Helicobacter pylori* in various disease states, such as peptic ulcer disease (PUD) and Mucosa Associated Lymphoid Tissue (MALT) lymphoma, was ground-breaking in the field of gastroenterology. Given the potentially dire clinical implications of chronic *H. pylori *infection, it is important to achieve complete eradication. More importantly, the rising prevalence of *H. pylori *antimicrobial resistance, similar to other pathogens world-wide, is of particular concern. Despite evidence supporting the growing threat of antimicrobial resistance, clinically, it is also important to survey just how much of the failed treatment is truly a reflection of resistance versus poor treatment adherence. In this report, we detail the case of a 64-year-old female who was previously given six treatment courses for persistent *H. pylori* infection. Successful eradication was achieved with rifabutin triple therapy consisting of high-dose amoxicillin and strict adherence monitoring by a clinical pharmacist. This case highlights the importance of patient education, medication reconciliation, and close monitoring to ensure successful treatment of persistent *H. pylori* infection.

## Introduction

*Helicobacter pylori* is a gram-negative, spiral-shaped, urease-producing microaerophilic bacterium that can be found on the gastric mucosal surface in approximately half of the world’s population. In the United States, the pooled *H. pylori* prevalence is estimated to be around 35.6% (95% CI 30.0%-41.1%) with higher predominance among patients with low socio-economic status [1]. Transmission can occur via fecal-oral, gastric-oral, oral-oral, or sexual routes, and once infected, a person can harbor the organism indefinitely unless treated [2]. Untreated *H. pylori* infection has been implicated in the development of chronic or atrophic gastritis, peptic ulcer disease, Mucosa Associated Lymphoid Tissue (MALT) lymphoma, and gastric carcinoma [3]. Most importantly, *H. pylori* is the only bacteria that has been classified as a group 1 carcinogen by the International Agency for Research on Cancer (IARC) [4]. 

For more than 20 years after its initial discovery in 1982, *H. pylori* has been treated with triple therapy that includes clarithromycin, amoxicillin, and high-dose proton-pump inhibitor (PPI). However, the rising clarithromycin resistance, reaching a prevalence of up to 15% in some regions, has forced practitioners to turn to other regimens [5]. Other factors complicating treatment success include treatment complexity, side-effects, cost, and patient understanding of the treatment regimen and its expected benefits [6]. We present a case of a 64-year-old female with persistent *H. pylori* infection despite administration of six treatment courses. Successful eradication of the infection was achieved with rifabutin triple therapy including high-dose amoxicillin and close treatment adherence monitoring by the clinic pharmacist.

## Case presentation

**Figure 1:**
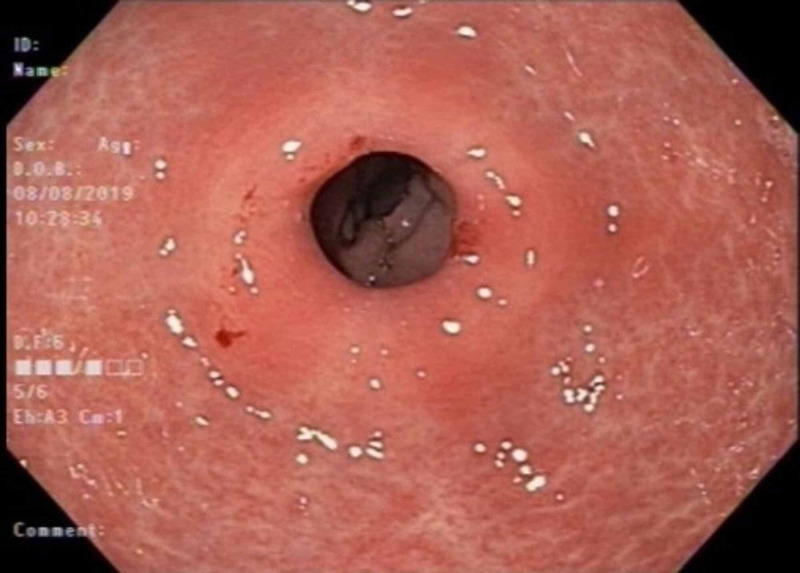
Endoscopic image revealing patchy erythematous antral mucosa

A 64-year-old African American female presented to the clinic with a four-month history of abdominal pain, bloating, and early satiety. She denied any unintentional weight loss or gastrointestinal bleeding. She denied use of nonsteroidal anti-inflammatory medications or opiates. Family history was negative for gastrointestinal cancers. She denied use of tobacco products, alcohol or illicit drugs. Her vital signs and physical exam were unremarkable except for epigastric tenderness. Lab work was pertinent for normocytic anemia with a hemoglobin (Hgb) of 11.8 g/dL. Electrolytes, renal, and hepatic function tests were within normal limits. Upper endoscopy showed gastric erythema and biopsies were positive for *H. pylori* infection (Figure 1).

**Figure 2:**
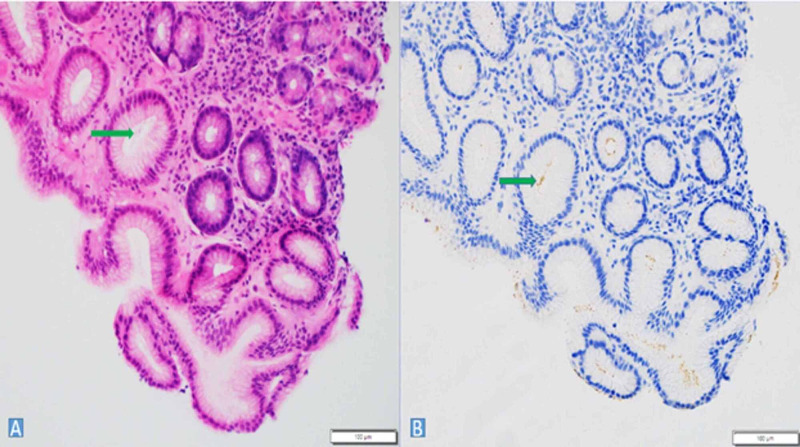
A- Gastric oxyntic mucosa reveals mild acute and chronic inflammatory infiltrate with rod/comma-shaped H. pylori organisms in the crypts (arrow). B- Confirmed by H. pylori IHC stain showing brown staining of organism in the crypts. Image courtesy of Dr. Nikhil Patel and Dr. Asad Ullah of Augusta University. IHC- Immunohistochemistry

A 14-day course of clarithromycin triple therapy was initially prescribed. The patient was then lost to follow-up and presented to the clinic one year later with similar symptoms. This time, she reported unintentional weight loss of 10 pounds in the past year. Repeat upper endoscopy revealed persistent *H. pylori* infection (Figure 2A, 2B).

Patient received the same clarithromycin triple therapy despite reported adherence to initial treatment. Confirmatory urea breath test revealed persistent infection. A 14-day course of bismuth quadruple therapy was then prescribed followed by 14-day levofloxacin triple therapy due to persistent infection. Repeat upper endoscopy with *H. pylori* culture and sensitivity revealed resistance to clarithromycin (Table 1).

**Table 1:** Helicobacter pylori culture and sensitivity S= SUSCEPTIBLE,   R= RESISTANT

Organism	Helicobacter pylori	
Antibiotic	MIC (mcg/mL)	Interpretation
Amoxicillin	0.12	S
Ciprofloxacin	2	S
Clarithromycin	>0.5	R
Metronidazole	64	S
Tetracycline	1	S

Rifampin triple therapy was subsequently prescribed but patient continued to remain symptomatic despite reported completion of treatment. Thereafter, a repeat upper endoscopy with *H. pylori *culture and sensitivity plus duodenal aspirates was performed which revealed small intestinal bacterial overgrowth (SIBO) and persistent *H. pylori *resistant to clarithromycin. Given the high susceptibility to amoxicillin 0.12mcg/mL (MIC), a high-dose dual regimen was prescribed. Symptoms partially improved for a few months but an indeterminate confirmatory urea breath test prompted another upper endoscopy, which was still positive for *H. pylori*. At this point, the patient had failed six treatment courses, despite reports of treatment adherence, which was documented by multiple providers. The decision was made to conduct a thorough medication fill history with the assistance of the clinical pharmacist. The clinical pharmacist, provider, and patient engaged in a lengthy phone conversation which led to the discovery of unintentional non-adherence to various prescribed regimens. For instance, patient was unaware that bismuth subsalicylate was an over-the-counter medication, which lead to an incomplete bismuth quadruple therapy treatment. In addition, it was discovered that the patient never received the rifampin as part of the rifampin triple therapy due to cost. It is highly likely that the patient failed the fourth prescribed regimen (levofloxacin triple therapy) despite reported strict medication compliance. After an exhaustive medication review and due to failure of traditionally prescribed *H. pylori *therapies, a salvage rifabutin triple therapy utilizing high dose amoxicillin 1 gram three times daily, and omeprazole 40 mg twice daily was prescribed (Table 2).

**Table 2:** The different Helicobacter pylori regimens in chronological order [7] QD- once daily, BID- twice daily, TID- three times daily *Medication patient did not take due to unawareness of it being an over the counter purchase. **Medication patient did not take due to cost.

	Regimens
1	Pantoprazole 40mg BID + Clarithromycin 500mg BID + Amoxicillin 1 gram BID
2	Pantoprazole 40mg BID + Clarithromycin 500mg BID + Amoxicillin 1 gram BID
3	Pantoprazole 40mg BID + Bismuth 2 tablets QID* + doxycycline 100mg BID + Metronidazole 500mg TID
4	Omeprazole 20mg BID + Levofloxacin 500mg QD + Amoxicillin 1 gram BID
5	Omeprazole 20mg BID + Amoxicillin 1 gram BID + Rifampin 300mg QD**
6	Omeprazole 40mg TID + Amoxicillin 1 gram TID
7	Omeprazole 40mg BID + Amoxicillin 1 gram TID + Rifabutin 300mg QD

The clinical pharmacist-provided patient counselling, mailed the patient a detailed medication calendar, and conducted phone interviews at least twice weekly to ensure treatment compliance (Table 3).

**Table 3:** Patient instructions by pharmacy team regarding schedule of medication administration *You received Rifabutin 150mg capsules, so you will take 2 capsules every morning with breakfast **You received Amoxicillin 500mg capsules, so you will take 2 capsules by mouth three times daily

30 minutes before breakfast (7 AM)	Breakfast (7:30 AM)	Afternoon (3:30 PM)	30 minutes before dinner (7 PM)	Bedtime (11:30 PM)
Omeprazole 40mg	Rifabutin 300mg* And Amoxicillin 1,000mg**	Amoxicillin 1,000mg**	Omeprazole 40mg	Amoxicillin 1,000mg**

After completion of this last treatment regimen, confirmatory urea breath test was finally negative and accompanied by resolution of her GI symptoms after a six-year battle to eradicate the infection. 

## Discussion

As evidenced by the case above, the eradication of *H. pylori* can be challenging on many fronts. Keys to successful eradication include not only appropriate antimicrobial treatment selection, but also patient's medication compliance.

A recent review by Fallone et al. reconciled the proposed antimicrobial treatment regimens of the Maastricht V/Florence report, American College of Gastroenterology (ACG), and Toronto Consensus [8]. For first-line treatment of *H. pylori *infections with unknown susceptibility, there are three generally accepted regimens: bismuth quadruple therapy, clarithromycin-based triple therapy, and concomitant therapy (triple therapy with metronidazole). In areas with resistance to both clarithromycin and metronidazole, bismuth quadruple therapy is preferred. Concomitant therapy is an option for patients that lack access to bismuth. All three recommending bodies agreed on an average 14-day course of treatment. Failure of a first-line therapy should prompt initiation of a salvage therapy, avoiding previously used antibiotics. All three groups agreed that the choice of second-line therapy should be bismuth quadruple therapy or levofloxacin triple therapy, depending on previously used antibiotics. Practitioners should ensure that patients are taking high dose PPIs for adequate acid suppression, in order to optimize antibiotic eradication of *H. pylori*. When re-evaluating the treatment approach, the Maastricht V/Florence report recommended an upper endoscopy with microbial cultures and sensitivity [9]. However, the reality of capturing sensitivity data may be impossible in more resource depleted areas or in patients that cannot tolerate an invasive procedure. Thus, high-dose dual therapy (amoxicillin and PPI) and rifabutin-based therapy are recommended as third and fourth-line treatments, depending on an individual’s previous treatment course. Resistance to antibiotics, particularly clarithromycin, remains a crucial factor affecting *H. pylori* eradication therapy [10]. Practitioner's familiarity with the available treatment regimens, local resistance rates, and the patient’s previous antibiotic use is paramount to determine initial therapy of choice to mitigate the impact of resistance. 

From a compliance standpoint, studies have shown that nearly 10% of patients treated with *H. pylori* eradication therapy will fail to take even 60% of the prescribed medications [11]. Strict adherence to treatment can impact outcomes positively as evidenced by a study performed by Graham et al. Eradication rates approached 96% in patients who took > 60% of the prescribed medications compared to 69% for those who took < 60% of the prescribed regimen [12]. When further dissecting into the contributing factors of treatment non-compliance, the regimen’s complexity, medication side effects, cost, and other patient-related factors (not wanting to take the medication, forgetfulness) are often cited. The FDA recently approved an all-in-one capsule (omeprazole magnesium, amoxicillin and rifabutin) as a therapy for *H. pylori*. This all-in-one capsule will hopefully improve patient compliance. Iatrogenic causes of treatment failure, such as repeated use of previously failed therapies, also contributes to poor eradication [13]. Medication reconciliation is necessary to avoid some of these pitfalls, but it requires an exhaustive history review. This investigative effort, much like with our patient, is severely limited by the time constraints of a typical 15-minute outpatient follow-up clinic visits in the United States.

There is limited research available on the impact of structured counselling and follow-up by the clinical pharmacist on rates of *H. pylori* eradication. Based on available evidence, there is a clear statistically significant improvement in *H. pylori* eradication employing this strategy [14]. Regarding our patient’s case, there was confusion about adherence to the components of the prescribed regimen and lack of communication regarding receipt of prescribed medications. These gaps were revealed during a lengthy phone interview reviewing the medication history of the last six years confirmed with the patient’s pharmacy dispensary history. As a result, when formulating her last treatment, the interdisciplinary team, consisting of the providers and the pharmacist, configured a detailed daily schedule, conducted intensive medication counselling including administration, possible side effects, and the importance of adherence, and provided multiple follow-up phone calls during the treatment. Although labor-intensive and time-consuming, these efforts led to the successful treatment of the patient’s persistent *H. pylori* infection. In retrospect, had there been clear patient instructions and counselling on the importance of strict adherence to treatment, reminder calendars, follow-up telephone calls, and detailed review of prior treatment regimens with every failed treatment course, successful *H. pylori* eradication could have been achieved earlier, possibly sparing the patient multiple medications and endoscopic procedures, which are both costly and invasive. This case highlights an interdisciplinary team’s vital role in ensuring successful *H. pylori* eradication and makes a case for further research to elucidate the full extent of clinical benefit of such an approach on a larger scale.

## Conclusions

Poor patient treatment compliance and increasing antimicrobial resistance are the two most important limiting factors in successfully eradicating *H. pylori* infection. Chronic *H. pylori* infection poses a significant public health threat as it has been linked to several serious pathological conditions including gastric carcinoma. We present a case of successful treatment of persistent *H. pylori* infection utilizing rifabutin triple therapy and a clinical pharmacist to ensure strict treatment adherence after six previous treatment courses. This case highlights the importance of appropriate medication selection and patient education as well as strict compliance monitoring to effectively reduce and eliminate cases of persistent *H. pylori*. Future studies should be conducted to investigate the impact of a dedicated clinical pharmacist as a member of the interdisciplinary team in the successful eradication of *H. pylori* infections. 

## References

[1] Hooi JKY, Lai WY, Ng WK (2017). Global prevalence of Helicobacter pylori infection: systematic review and meta-analysis. Gastroenterology.

[2] Parikh NS, Ahlawat R (2020). Helicobacter Pylori. https://www.ncbi.nlm.nih.gov/books/NBK534233/.

[3] Kao C-Y, Sheu B-S, Wu J-J (2016). Helicobacter pylori infection: an overview of bacterial virulence factors and pathogenesis. Biomed J.

[4] IARC Working Group on the Evaluation of Carcinogenic Risks to Humans (1994). Schistosomes, Liver Flukes and Helicobacter pylori.

[5] Savoldi A, Carrara E, Graham DY, Conti M, Tacconelli E (2018). Prevalence of antibiotic resistance in Helicobacter pylori: a systematic review and meta-analysis in World Health Organization regions. Gastroenterology.

[6] O'Connor JP, Taneike I, O'Morain C (2009). Improving compliance with helicobacter pylori eradication therapy: when and how?. Therap Adv Gastroenterol.

[7] Chey WD, Leontiadis GI, Howden CW, Moss SF (2017). ACG Clinical Guideline: treatment of Helicobacter pylori infection.. Am J Gastroenterol.

[8] Fallone CA, Moss SF, Malfertheiner P (2019). Reconciliation of recent Helicobacter pylori treatment guidelines in a time of increasing resistance to antibiotics. Gastroenterology.

[9] Malfertheiner P, Megraud F, O'Morain CA (2017). Management of Helicobacter pylori infection-the Maastricht V/Florence Consensus Report. Gut.

[10] Tang Y, Tang G, Pan L, Zhu H, Zhou S, Wei Z (2020). Clinical factors associated with initial Helicobacter pylori eradication therapy: a retrospective study in China. Sci Rep.

[11] Lee M, Kemp JA, Canning A, Egan C, Tataronis G, Farraye FA (1999). A randomized controlled trial of an enhanced patient compliance program for Helicobacter pylori therapy. Arch Intern Med.

[12] Graham D, Lew G, Malaty H (1992 ). Factors influencing the eradication of Helicobacter pylori with triple therapy. Gastroenterology.

[13] Li H, Liang X, Chen Q, Zhang W, Lu H (2018). Inappropriate treatment in Helicobacter pylori eradication failure: a retrospective study. Scand J Gastroenterol.

[14] Al-Eidan FA, McElnay JC, Scott MG, McConnell JB (2002). Management of Helicobacter pylori eradication--the influence of structured counselling and follow-up. Br J Clin Pharmacol.

